# A case of left frontal high-grade glioma diagnosed during pregnancy

**DOI:** 10.1186/s40981-017-0090-9

**Published:** 2017-04-26

**Authors:** Kotoe Kamata, Risa Fukushima, Minoru Nomura, Makoto Ozaki

**Affiliations:** Department of Anesthesiology, Tokyo Women’s Medical University, 8-1 Kawada-cho, Shinjuku-ku, Tokyo, 162-8666 Japan

**Keywords:** High-grade glioma, Pregnancy, Therapeutic strategy, Decision-making, Awake craniotomy

## Abstract

**Background:**

As pregnancy accelerates glioma growth, therapeutic abortion has been recommended prior to tumor resection. Additionally, it has also been suggested that the extent of glioma resection is closely correlated with patient survival.

**Case presentation:**

A 162-cm, 61.4-kg, 30-year-old, right-handed primigravida was referred to our institution at 21 weeks gestation to obtain a second opinion. At 18 weeks gestation, the patient developed new-onset generalized convulsive seizures (GCSs), which were poorly controlled by anticonvulsant polytherapy, early in the second trimester. A 6-cm lesion located in her left frontal supplementary motor area (SMA) was suspected as a grade III glioma, classified according to the World Health Organization (WHO) guidelines. Due to the limited evidence on the use of adjuvant therapy during pregnancy, tumors causing neurological symptoms and seizures must be treated, in order to stabilize the maternal condition and enable a safe birth. In the case of pregnant patients, awake craniotomy using intraoperative magnetic resonance imaging (iMRI) is considered advantageous, achieving gross total resection with a reduction of direct cortical stimulation, which may induce seizure, and so reducing fetal exposure to anesthetics. The “Asleep-Awake-Asleep” technique was performed at 27 weeks and 2 days gestation. As use of propofol in pregnant patients is prohibited, general anesthesia was maintained through administration of sevoflurane and remifentanil until the first scan of iMRI, and was subsequently re-induced with dexmedetomidine when tumor removal had been accomplished. A supraglottic airway (SGA) was used until the patient’s cranium was opened. There were no complications during either the procedure or the post-operative period. At 35 weeks gestation, the patient delivered a healthy baby of 2317 g. Pathological examination of the patient, revealed an anaplastic astrocytoma, thus radiotherapy and chemotherapy began 2 months post-delivery. There is no evidence of tumor recurrence in the patient and the child did not show any medical or developmental concerns at the point of the 17-month follow-up.

**Conclusions:**

Since evidence on the use of adjuvant therapy during pregnancy is limited, extensive resection with functional monitoring is recommended if a brain tumor is presumed to be malignant. Awake craniotomy is considered advantageous to pregnant patients because subjective movement preserves the patient’s motor function and reduces fetal exposure to anesthetics. Therefore, providing multidisciplinary discussion takes place within the decision-making process, as well as careful perioperative preparation, awake craniotomy should be considered, even in the case of pregnant patients.

## Background

Treatment of malignant glioma in pregnant patients presents several clinical challenges, because it involves multi-factorial conditions. It has been suggested that the extent of glioma resection is significantly correlated with patient survival [1]. Thus, maximal surgical resection with a minimal risk of teratogenicity should be attained in pregnant women, providing the fetus is viable at the time of planned neurosurgery.

## Case presentation

### Pre-operative course

**Fig. 1:**
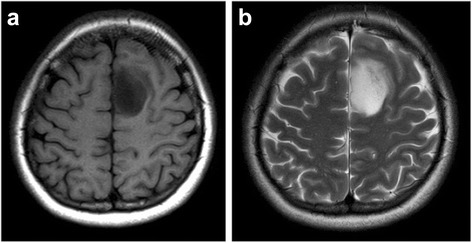
Axial sections of magnetic resonance imaging scan performed 3 days before operation. T1-weighted image as hypointense (**a**) and T2-weighted image as hyperintense (**b**); mass is located in the left superior frontal gyrus

A 162-cm, 61.4-kg, 30-year-old, right-handed primigravida was referred to our institution at 21 weeks gestation to obtain a second opinion; although, the fetus did not show signs of growth retardation, therapeutic abortion had been put forward by her previous doctor. MRI revealed a 6-cm mass lesion on the T1-weighted image as hypointense and on the T2-weighted image (T2WI) as hyperintense in her left frontal SMA, which had been misdiagnosed 8 years earlier [Fig. 1]. Anti-epileptic therapy with lamotrigine (100 mg, daily) and levetiracetam (1000 mg, daily) was started following the first GCS, which developed at 18 weeks gestation. Persistent morning sickness was treated with metoclopramide (15 mg, daily). A multidisciplinary conference with the patient and her family was held at 26 weeks gestation and a therapeutic strategy for tumor removal, to be performed prior to delivery, was proposed. The lesion of the patient, a suspected grade III glioma according to the WHO classification guidelines, was located within the SMA of her dominant hemisphere. Uncontrollable GCS suggested rapid growth of the brain tumor. However, therapeutic abortion could not be performed as the patient had already exceeded the 22-week gestation mark. Against our proposal, the patient selected awake craniotomy, thus allowing her to complete a full-term pregnancy, with adjuvant therapy added if it was required. Awake craniotomy using the “Asleep-Awake-Asleep” technique for resection of the left frontal SMA glioma was scheduled for 27 weeks and 2 days gestation. The patient was evaluated as American Society of Anesthesiologists Physical Status Class 2.

Despite increasing the daily dose of lamotrigine to 150 mg, the patient’s GCS did not cease. Add-on therapy of 10 mg of clobazam was administered 1 day before surgery. In order to prevent maternal hypotension caused by anesthesia, the patient received aspiration prophylaxis with intravenous ranitidine 50 mg and metoclopramide 10 mg, along with 500 mL of 6% hydroxyethyl starch (70/0.5) 1 h before anesthesia induction. Preparations were also made for emergency caesarean delivery in case of maternal or fetal deterioration.

### Awake craniotomy

In the operating room, the patient was positioned supine and a wedge was placed under the right buttock to prevent aortocaval compression. The obstetrician commenced fetal heart rate (FHR) monitoring and confirmed reassuring fetal status (RFS). Antiemetic prophylaxis of dexamethasone 4.95 mg was given and an arterial catheter was inserted. Following pre-oxygenation, rapid sequence induction with cricoid pressure was carried out using fentanyl 125 μg and thiopental 375 mg. The patient’s airway was secured through use of i-gel® #3 (INTERSURGICAL, UK) and a gastric tube was placed. Ventilation was adjusted to maintain the patient’s normal PaCO_2_ at 31 mmHg. General anesthesia was maintained with 40% oxygen in air, sevoflurane and remifentanil at 0.3 μg/kg/min. Sevoflurane concentration was adjusted to attain the Bispectral Index® (Covidien, Dublin, Ireland) value of 50. Scalp blocks and infiltration analgesia were provided with 40 mL of 0.3% ropivacaine and 10 mL of 1% lidocaine with 0.01% epinephrine, respectively. Five grams of glucose was given for maternal hypoglycemia (blood glucose level was 82 mg/dL) after induction. As the patient’s base excess level gradually decreased from −4.9 to −6.8, maternal acidosis was suspected to be progressing, so sodium bicarbonate 10.5 g was administered to compensate for this (pH ranged 7.35–7.41).

The first iMRI was performed following the left frontal craniotomy. All anesthetics were ceased and the patient regained consciousness 137 min after induction took place. The patient did not agitate. Neither coughing nor aspiration was observed. FHR also recovered to RFS. Tumor margin dissection was implemented in accordance with an updated neuro-navigation system and iMRI. During the removal of the posterior half of the tumor, the patient’s subjective movement was weakened without any deterioration of motor-evoked potential (MEP) responses. Following main mass resection, extensive removal of the T2WI area was carried out, as the area extended to the next gyrus through U-fiber in the white matter. Seizure was not observed and a high arousal condition was maintained. Nausea was controlled through 3 mg of granisetron to prevent vomiting. For pain control, 13 mL in total of 1% lidocaine with 0.01% epinephrine was used. The patient did once complain of abdominal pain due to fetal movement, however FHR remained 130–155 bpm without deceleration.

**Fig. 2:**
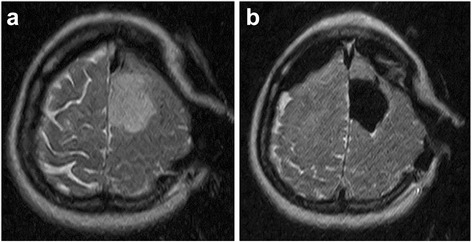
T2-weighted scout images of intraoperative magnetic resonance imaging scan. The first intraoperative magnetic resonance imaging was performed after craniotomy (**a**). Extent removal of the left frontal tumor was confirmed before surgical site closure (**b**)

Complete removal of T2WI area was confirmed by the second iMRI; obtained 87 min into the awake phase [Fig. 2]. Conscious sedation was provided using dexmedetomidine, which was started at 1.0 μg/kg/h and continued at 0.7 μg/kg/h 20 min thereafter. During surgical site closure, fentanyl 225 μg and droperidol 100 μg were given. From the beginning of the awake phase, maternal blood pressure remained within 20% of the baseline level without pharmacological manipulation. However, during the initial asleep phase, 4 boluses, each of phenylephrine 50 μg and ephedrine 4 mg, were administered. FHR was stable at 130 bpm without variability when the patient was sedated. A total of 2350 mL bicarbonate Ringer’s solution was infused. Total urine output was 540 mL and estimated blood loss was 107 mL. The total duration of the surgery and anesthesia was 241 and 291 min, respectively.

### Post-operative course

**Fig. 3:**
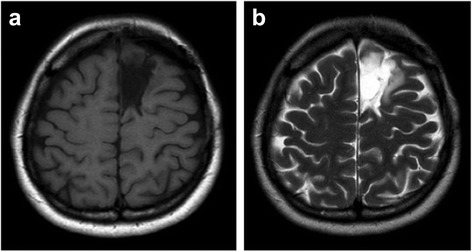
Axial sections of magnetic resonance imaging scan performed 11 months after awake craniotomy. T1-weighted image (**a**) and T2-weighted image (**b**) show no evidence of tumor recurrence

The patient developed no new neurological deficits following the operation. A healthy baby of 2317 g was vaginally delivered at 35 weeks and 2 days gestation. As pathological examination revealed an anaplastic astrocytoma (WHO grade III), radiotherapy and chemotherapy began 2 months after delivery. There was no evidence of tumor recurrence in the patient and the child did not show any medical or developmental concerns at the point of the 17-month follow-up [Fig. 3].

### Discussion

#### Therapeutic strategy for pregnant patients with high-grade glioma

An important lesson learned from this pregnant case is the caution required in developing a management strategy for eloquent high-grade gliomas. The guidelines for the diagnosis and treatment of gliomas, released by the European Association for Neuro-Oncology, present the management options for newly diagnosed malignant glioma as resection or biopsy, followed by radiotherapy or chemotherapy (or combined modality treatment) [2]. In the case of malignant tumors in pregnant patients, neurosurgical intervention is recommended regardless of gestational age; however, the 32 weeks gestation point is generally used as the cutoff [3, 4]. In our patient, progressive growth of the left frontal SMA glioma caused GCSs, which required multidrug therapy with anticonvulsants. As radiological examination using contrast reagent was avoided, malignant transformation could not be ruled out. Due to the gestational age therapeutic abortion was no longer an alternative (in Japan the limit for legal abortion is 22 weeks). Therefore, we selected a therapeutic strategy of tumor removal to be performed prior to delivery. In fact, pregnancy has been shown to accelerate glioma growth [5]. However, there is a paucity of information on the long-term development of newborns from mothers receiving anticonvulsants, chemotherapeutics, or radiation during pregnancy [6, 7]. As Nitta et al. reported, maximum tumor resection should be aimed for in malignant glioma treatment, as the extent of tumor resection is a strong prognosis factor irrespective of tumor subtypes. They also suggested that patients with a high extent of resection could be safely monitored without the need for post-operative chemotherapy and radiotherapy [1]. For patients in the second and early third trimesters, it is safe to perform a craniotomy initially and allow the patient to complete a full-term pregnancy [8].

Extensive resection with neurophysiological monitoring is considered to be the best way to preserve patient motor function. Maximum resection of a T2WI lesion can be achieved using an updated neurosurgical navigation system and iMRI [9], which is suitable for pregnant patients [10]. In the field of neuro-anesthesia, propofol is a commonly used sedative, as it is less disturbing to MEP recording than volatile agents [11]. However, two cases have been reported of prolonged propofol general anesthesia for neurosurgery during pregnancy (14–18 h), resulting in mild metabolic acidosis after 11 and 10 h, respectively. The reports suggest that propofol should not be used for very long procedures [12]. It is actually stated by the manufacturer that propofol use is contraindicated during pregnancy in several countries, including Japan. In this situation, sevoflurane, which is known to deteriorate intraoperative neurophysiological monitoring, becomes an alternative. A multidisciplinary conference was held with neurosurgeons, obstetricians, nursing staff, and neuro-anesthesiologists, along with the patient and her family, to discuss the most suitable surgical intervention/anesthesia for this particular case. Considering the detrimental effects of tumor progression, as well as both the pharmacological limitations and unfavorable effects of general anesthesia on the patient and the fetus, we reached the unanimous decision to conduct awake craniotomy early in the third trimester; and so enable a full term birth. Adjuvant therapy was planned to commence once the lactation period was complete. Considering the adverse effects of maternal hypoxia on a fetus, we also prepared a secondary plan of converting to brain biopsy under general anesthesia to achieve histopathological diagnosis. While awake craniotomy in pregnant patients is still challenging [10, 13, 14], our case clearly shows that substitution of the patient’s subjective movement for MEP, was successful in confirming the preservation of motor function. Furthermore, minimization of exposure to anesthetics is also advantageous to the fetus.

#### Pitfalls of anesthetic management of awake craniotomy for parturient

We selected the “Asleep-Awake-Asleep” technique to minimize the patient’s physiological and psychological stress; as the patient need only be awake for the intraoperative testing portion. In general, SGA is preferred to endotracheal intubation for awake craniotomies because SGA preserves the patient’s speech function and suppresses cardiorespiratory fluctuations [15]. Contrary to common belief, during pregnancy gastric emptying is not significantly altered [16], and the volume and acidity of gastric secretions remain unchanged [17, 18]. Indeed, SGAs have been successfully used for airway management in pregnant cases [19, 20]. However, we should remember that pregnant women are more likely to experience both symptomatic and silent regurgitation. According to our experience, aspiration prophylaxes of antiemetics as well as acid aspiration have been shown to be effective in preventing intraoperative vomiting of pregnant patients undergoing awake craniotomy. Furthermore, the patient stays in a head-up position and the gastric contents should be well drained during the initial “Asleep” phase.

Another important issue is the type of anesthetics. Propofol and dexmedetomidine are commonly used in awake craniotomy, but as mentioned before, propofol is prohibited for pregnant use in Japan. Dexmedetomidine use for the patients with a secured airway is also “off-label”. Therefore, in our case sevoflurane was administered to the patient until the craniotomy had been completed. In obstetric and neurosurgical anesthesia, volatile anesthetics are favored: in the latter for the reduction in cerebral metabolic rate with minimal impact on intracranial pressure [21]. On the other hand, volatile anesthetics have a tocolytic effect. Since the minimum alveolar concentration is reduced during pregnancy, depth of anesthesia monitoring is considered necessary for avoiding sevoflurane overuse. While FHR monitoring could potentially alert the anesthesiologists to the development of fetal hypoxia, allowing restoration of blood pressure or anesthesia depth, FHR monitoring during non-obstetric surgery remains controversial. In addition, consideration should also be given to the use of scalp infiltration, as noxious stimulation can elicit a hypertensive response or emotional intolerance during the awake craniotomy procedure.

## Conclusions

Since the extent of tumor resection of malignant glioma is a strong prognosis factor, awake craniotomy should be considered as a therapeutic option for pregnant patients; when the glioma is in or adjacent to the eloquent area. Although both anesthetic and airway device options are limited for use on pregnant patients, the “Asleep-Awake-Asleep” technique using sevoflurane and SGA described earlier is regarded as a useful and less invasive option.

## Abbreviations

FHR: Fetal heart rate
GCS: Generalized convulsive seizure
iMRI: Intraoperative magnetic resonance imaging
MEP: Motor-evoked potential
RFS: Reassuring fetal status
SGA: Supraglottic airway
SMA: Supplementary motor area
T2WI: T2-weighted image
WHO: World Health Organization

## References

[1] Nitta M, Muragaki Y, Maruyama T, Ikuta S, Komori T, Maebayashi K (2015). Proposed therapeutic strategy for adult low-grade glioma based on aggressive tumor resection. Neurosurg Focus..

[2] Weller M, van den Bent M, Hopkins K, Tonn JC, Stupp R, Falini A (2014). EANO guideline for the diagnosis and treatment of anaplastic gliomas and glioblastoma. Lancet Oncol..

[3] Wlody DJ, Weems L, Cotterell JE, Young WL (2010). Anesthesia for neurosurgery in the pregnant patient. Cotterell and Young's Neuroanesthesia.

[4] Lynch JC, Gouvêa F, Emmerich JC, Kokinovrachos G, Pereira C, Welling L (2011). Management strategy for brain tumour diagnosed during pregnancy. Br J Neurosurg..

[5] Pallud J, Mandonnet E, Deroulers C, Fontaine D, Badoual M, Capelle L (2010). Pregnancy increases the growth rates of World Health Organization grade II gliomas. Ann Neurol..

[6] Tomson T, Landmark CJ, Battino D (2013). Antiepileptic drug treatment in pregnancy: changes in drug disposition and their clinical implications. Epilepsia..

[7] Zwinkels H, Dörr J, Kloet F, Taphoorn MJB, Vecht CJ (2013). Pregnancy in women with gliomas: a case-series and review of the literature. J Neurooncol..

[8] Elwatidy S, Jamjoom Z, Elgamal E, Abdelwahab A (2011). Management strategies for acute brain lesions presenting during pregnancy: a case series. Br J Neurosurg..

[9] Muragaki Y, Iseki H, Maruyama T, Tanaka M, Shinohara C, Suzuki T (2011). Information-guided surgical management of gliomas using low-field-strength intraoperative MRI. Acta Neurochir Suppl..

[10] Handlogten KS, Sharpe EE, Brost BC, Parney IF, Pasternak J (2015). Dexmedetomidine and mannitol for awake craniotomy in a pregnant patient. Anesth Analg..

[11] Sloan TB, Jäntti V, Nuwer MR (2008). Anesthetic effects on evoked potentials. Intraoperative Monitoring of Neural Function: Handbook of Clinical Neurophysiology.

[12] Sethuraman M, Neema PK, Rathod RC (2007). Prolonged propofol infusion in pregnant neurosurgical patients. J Neurosurg Anesthesiol..

[13] Abd-Elsayed AA, Díaz-Gómez J, Barnett GH, Kurz A, Inton-Santos M, Barsoum S (2013). A case series discussing the anaesthetic management of pregnant patients with brain tumours. F1000Research.

[14] Meng L, Han SJ, Rollins MD, Gelb AW, Chang EF. Awake brain tumor resection during pregnancy: decision making and technical nuances. J Clin Neurosci. 2015. doi: 10.1016/j.jocn.2015.08.021. 10.1016/j.jocn.2015.08.021 26498092

[15] Dinsmore J, Brambrink AM, Kirsch JR (2012). Challenges during anaesthesia for awake craniotomy. Essentials of Neurosurgical Anesthesia & Critical Care.

[16] Wong CA, Loffredi M, Ganchiff JN, Zhao J, Wang Z, Avram MJ (2002). Gastric emptying of water in term pregnancy. Anesthesiology..

[17] Van Thiel DH, Gavaler JS, Joshi SN, Sara RK, Stremple J (1977). Heartburn of pregnancy. Gastroenterology..

[18] O'Sullivan GM, Bullingham RE (1984). The assessment of gastric acidity and antacid effect in pregnant women by a non-invasive radiotelemetry technique. Br J Obstet Gynaecol..

[19] Preston R (2001). The evolving role of the laryngeal mask in obstetrics. Can J Anaesth..

[20] Berger M, Corso RM, Piraccini E, Agnoletti V, Valtancoli E, Gambale G (2011). The i-gel in failed obstetric tracheal intubation. Anaesth Intensive Care..

[21] Koerner IP, Brambrink AM (2006). Brain protection by anesthetic agents. Curr Opin Anaesthesiol..

